# Supplementary material for: UFD-2 is an adaptor-assisted E3 ligase targeting unfolded proteins
Source: Nat Commun. 2018 Feb 2;9:484. doi: 10.1038/s41467-018-02924-7 (PMC5797217; doi:10.1038/s41467-018-02924-7)
Supplement: Supplementary file 1 — Supplementary Information [file 41467_2018_2924_MOESM1_ESM.pdf]

SUPPLEMENTARY FIGURES

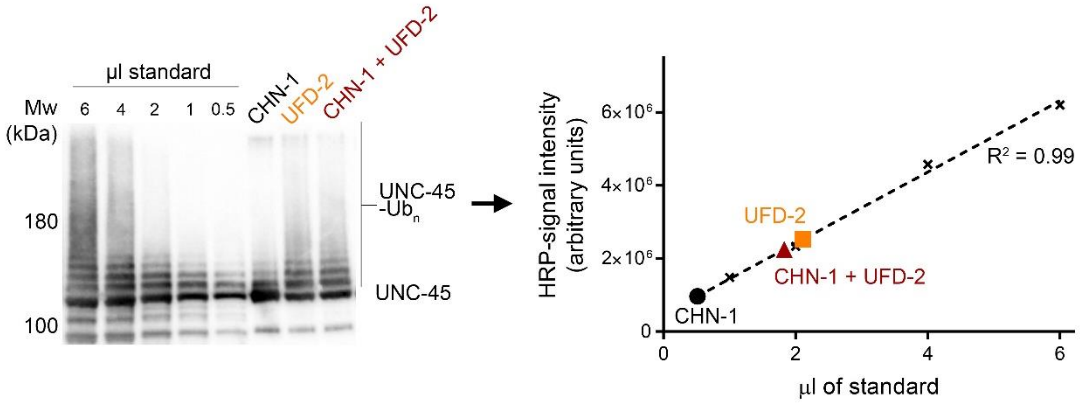

Supplementary Figure 1. Quantification of ubiquitination reactions showing the linear range of the signal.

Left panel: anti-UNC-45 Western blot showing a dilution series (µl of standard reaction) of a ubiquitination reaction containing wild-type UNC-45 and UFD-2, and reactions containing the indicated E3 ligase(s). Right panel: Quantification of ubiquitinated UNC-45. Known amounts of the ubiquitination reaction containing wild-type UNC-45 and UFD-2 (µl of standard) were plotted against the quantified ubiquitination signal to generate a standard curve. All reactions are in the linear range of detection.

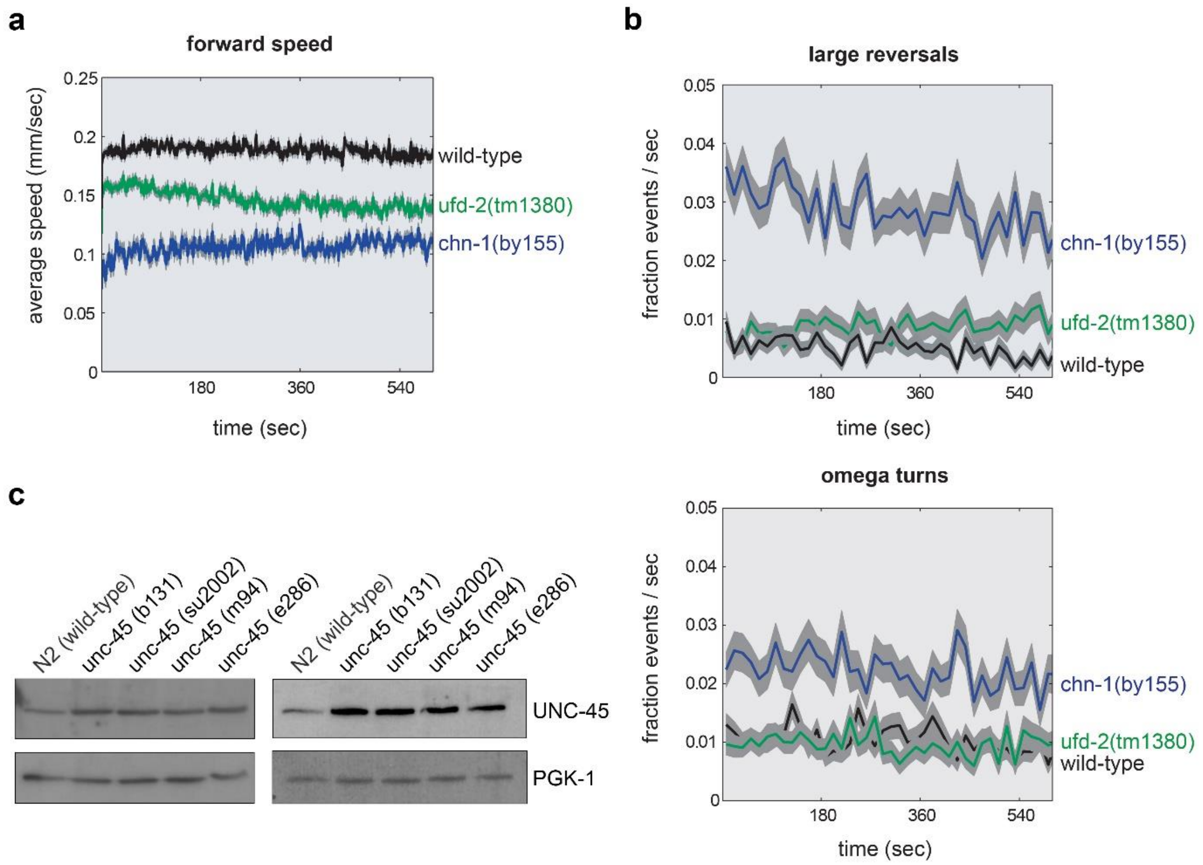

Supplementary Figure 2. Motility assays of *C. elegans* strains

(**a**) Average speed of 60 to 100 young adult N2 (wild-type), *chn-1(by155)* or *ufd-2(tm1380)* worms as monitored on NGM plates over 600 sec without food. Each strain was assayed three times. (**b**) *chn-1(by155)* worms display a distinct reorientation behavior as illustrated by a higher frequency of large reversals and omega turns compared to wild-type and *ufd-2(tm1380)* worms. Traces of behavioral time courses show mean and shaded s.e.m. of all animals. (**c**) Western blot analysis showing the protein levels of endogenous UNC-45 variants in the indicated *C. elegans ts*-strains grown at 16°C. PGK-1 is shown as a loading control.

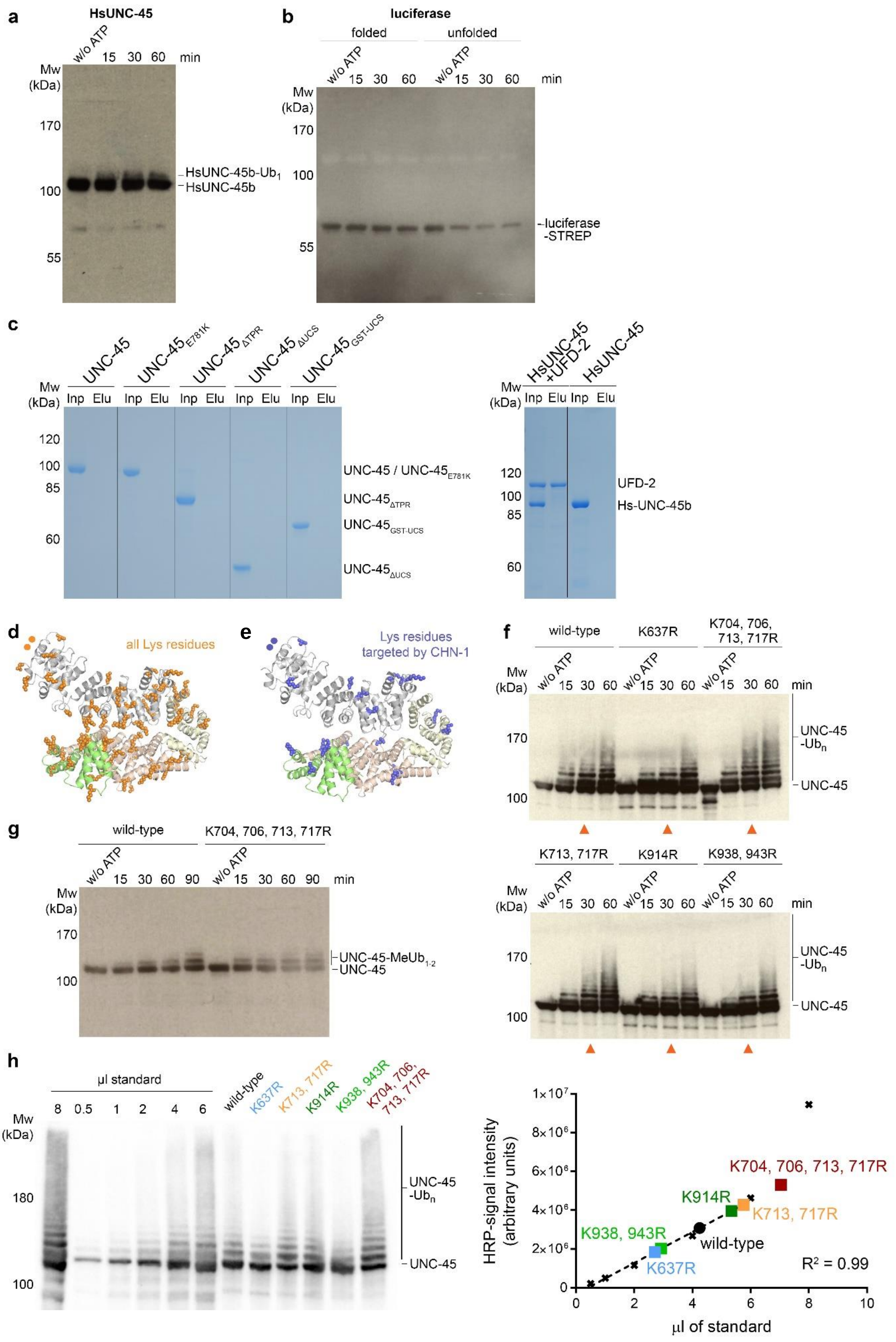
a
HsUNC-45
w/o ATP
15 30 60 min
Mw (kDa)
170
100
55
HsUNC-45b-Ub1
HsUNC-45b
b
luciferase
folded
unfolded
w/o ATP 15 30 60
w/o ATP 15 30 60 min
Mw (kDa)
170
100
55
luciferase -STREP
c
UNC-45
UNC-45E781K
UNC-45ΔTPR
UNC-45ΔUCS
UNC-45GST-UCS
Mw (kDa)
Inp Elu Inp Elu Inp Elu Inp Elu Inp Elu
120
100
85
60
UNC-45 / UNC-45E781K
UNC-45ΔTPR
UNC-45GST-UCS
UNC-45ΔUCS
HsUNC-45 +UFD-2
HsUNC-45
Inp Elu Inp Elu
120
100
85
60
UFD-2
Hs-UNC-45b
d
all Lys residues
e
Lys residues targeted by CHN-1
f
wild-type
K637R
K704, 706, 713, 717R
w/o ATP 15 30 60
min
Mw (kDa)
170
100
UNC-45 -Ubn
UNC-45
K713, 717R
K914R
K938, 943R
g
wild-type
K704, 706, 713, 717R
w/o ATP 15 30 60 90
min
Mw (kDa)
170
100
UNC-45-MeUb1-2
UNC-45
h
µl standard
8 0.5 1 2 4 6
wild-type
K637R
K713, 717R
K914R
K938, 943R
K704, 706, 713, 717R
Mw (kDa)
180
100
UNC-45 -Ubn
UNC-45
HRP-signal intensity (arbitrary units)
1×10^7
8×10^6
6×10^6
4×10^6
2×10^6
0
0 2 4 6 8 10
µl of standard
K704, 706, 713, 717R
K914R
K713, 717R
K938, 943R
wild-type
K637R
R² = 0.99

Supplementary Figure 3. Analysis of UFD-2 substrate specificity

Ubiquitination of HsUNC-45b (**a**) and luciferase (**b**) by UFD-2. Reactions were incubated for 15, 30 and 60 min in the presence of ATP and the control reaction for 60 min without ATP. Reactions were analyzed by anti-HsUNC-45b and anti-STREP Western blot respectively. (**c**) Left panel: Control for PD experiments shown in **Fig. 4e**, demonstrating the UNC-45 proteins do not interact with the resin. Right panel: PD study with HsUNC-45b and UFD-2 and the corresponding control showing that the two proteins do not interact. (**d**) Cartoon representation of UNC-45 (PDB code: 4i2z) with TPR, central, neck and UCS domains colored in green, orange, yellow and grey respectively. All lysine residues are shown as orange spheres. (**e**) UNC-45 structure highlighting lysine residues modified by CHN-1 in blue. (**f**) Ubiquitination of different UNC-45 KR point mutants. The reactions were incubated for 15, 30 and 60 min with ATP and analyzed by anti-UNC-45 Western blot. (**g**) Time course analysis (15, 30, 60, 90 min) of UNC-45 KR_{canyon} ubiquitination using MeUb as a control for testing the poly-ubiquitination of the substrate. (**h**) Left panel: anti-UNC-45 Western blot showing dilution series (µl of standard reaction) of a ubiquitination reaction containing wild-type UNC-45 and UFD-2, and reactions containing the indicated version of UNC-45 protein and UFD-2. Right panel: Quantification of ubiquitinated UNC-45. Known amounts of the ubiquitination reaction containing wild-type UNC-45 (µl of standard) were plotted against the quantified ubiquitination signal to generate a standard curve using the first five data points. The reaction containing UNC-45 K704, 706, 713, 717R is close to the limit of the linear range, but still clearly shows an increase in ubiquitination under the applied Western blot conditions as the reference reaction containing wild-type UNC-45 is definitively in the linear range of detection.

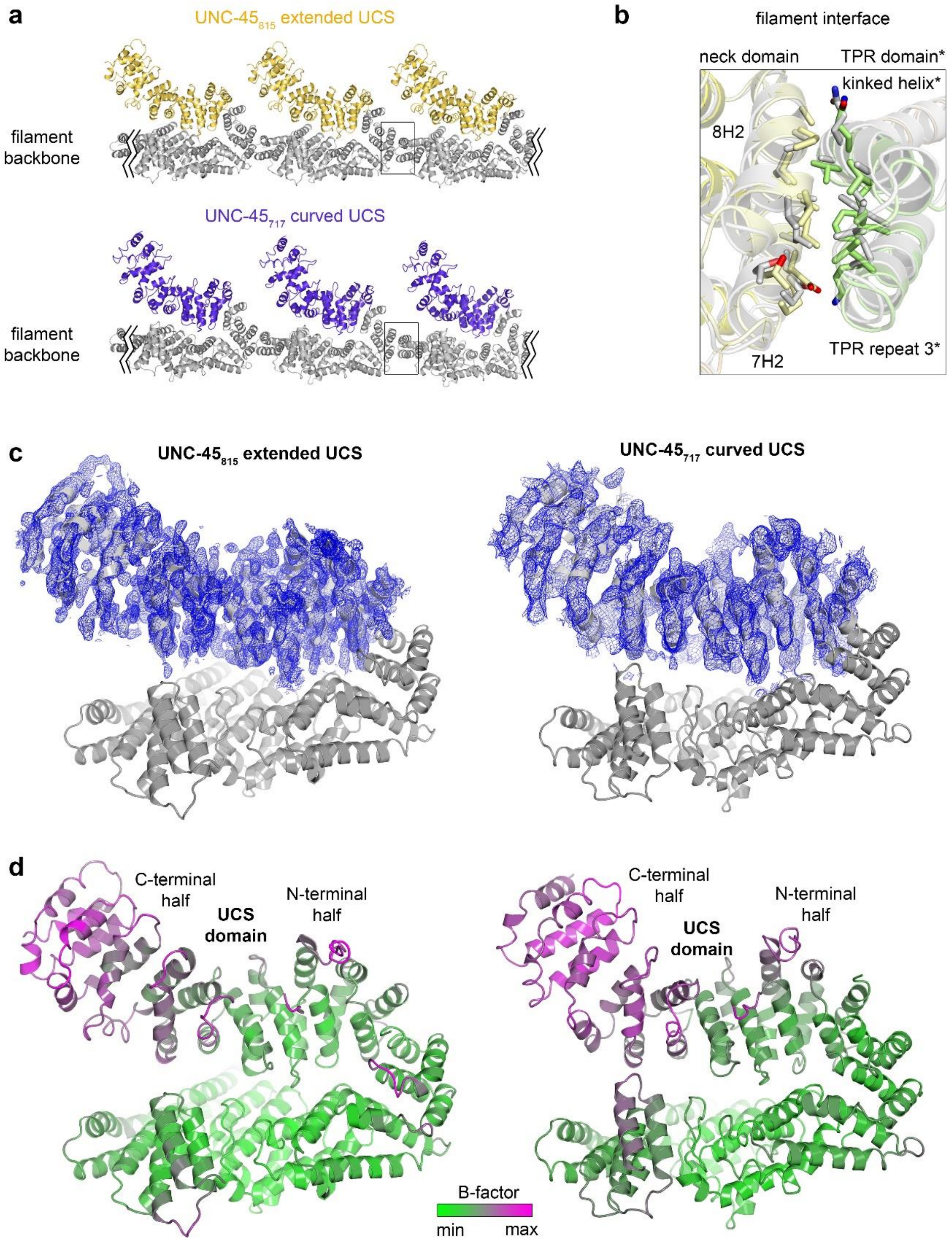

Supplementary Figure 4. Protein filaments observed in distinct UNC-45 crystal lattices

(**a**) Cartoon representation showing the UNC-45 filament that is assembled from the same backbone in the UNC-45_{717} and UNC-45_{815} structures. In analogy to UNC-45_{815}, the UNC-45_{717} molecule formed infinite protein chains in the crystal lattice

using the same oligomerization interface. The backbone of the filament composed of the TPR, central and neck domain superimpose well (rms deviation of all Cα atoms about 1.0 Å) suggesting that chain formation is a common property of the two UNC-45 isoforms. The differently orientated UCS domains are colored blue and yellow respectively. (**b**) Inset shows an overlay of the filament interfaces observed for the UNC-45_{717} (grey) and UNC-45_{815} (colored) structures. Two helices from the neck domain (7H2 and 8H2) interact with the kinked helix and helix B of TPR repeat 3 of the crystallographic neighbor (indicated by asterisk). Residues important for filament formation (shown in stick mode) further illustrate that the different UCS conformations are part of otherwise almost identical protein filaments (**c**) 2m*F*o-D*F*c electron density maps (blue) are shown for the UCS domains of UNC-45_{815} (left, 2.9 Å resolution) and UNC-45_{717} (right, 3.8 Å resolution). The maps are contoured at 1.0 σ and were generated using PHENIX. (**d**) B-factor plots: Ribbon presentations of UNC-45_{815} (left) and UNC-45_{717} (right) with mapped B-factors using a green-to-magenta color ramp ranging from 30-250 $Å^2$ and 80-310 $Å^2$, respectively.

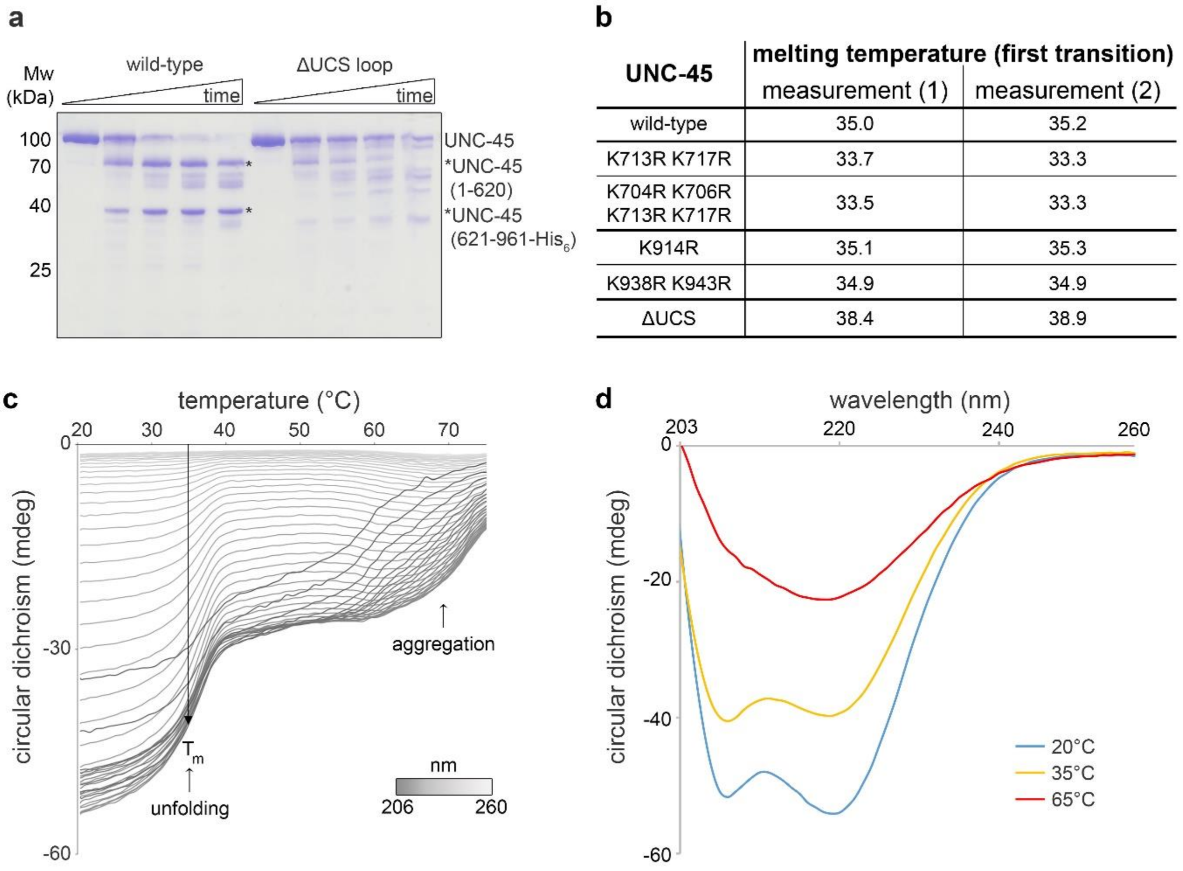

UNC-45	melting temperature (first transition)	
	measurement (1)	measurement (2)
wild-type	35.0	35.2
K713R K717R	33.7	33.3
K704R K706R K713R K717R	33.5	33.3
K914R	35.1	35.3
K938R K943R	34.9	34.9
ΔUCS	38.4	38.9

Supplementary Figure 5. Limited proteolysis and CD spectroscopy of UNC-45 variants

(**a**) Time course analysis of trypsin digests (0, 5, 15, 30 and 60 min incubation) of wild-type UNC-45, showing the formation of a 70 kDa and a 40 kDa fragment corresponding to residues 1-620 and 621-961, respectively. The characteristic cleavage products are not observed in digests of an UNC-45 mutant lacking the UCS loop ($UNC\text{-}45_{\Delta UCS\ loop}$). (**b**) Melting temperatures (T_m) of UNC-45 variants, as determined by CD spectroscopy in two independent measurements. (**c**) Melting curves recorded from 200-260 nm (1 nm steps) at increasing temperature from 20 to 75°C (1°C steps). The T_m value is determined as the temperature at the indicated inflection point of the recorded curves, characterizing the protein unfolding step. (**d**) Individual CD curves showing the transition from folded (20°C) to partially folded (35°C) and fully unfolded/aggregated (65°C) protein.

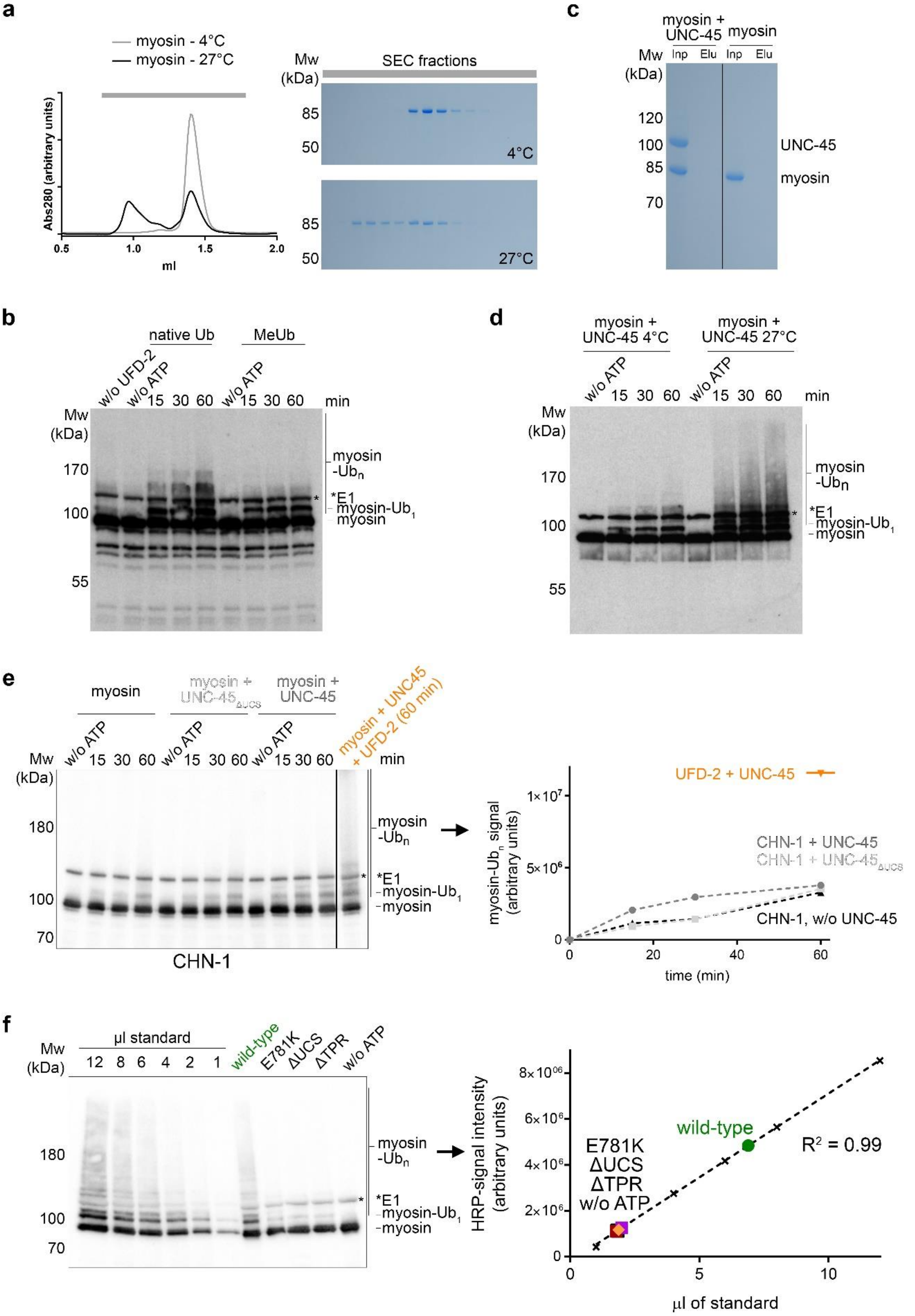
a
myosin - 4°C
myosin - 27°C
Abs280 (arbitrary units)
0.5
1.0
1.5
2.0
ml
Mw (kDa)
SEC fractions
85
50
4°C
85
50
27°C
b
native Ub
MeUb
w/o UFD-2
w/o ATP
15 30 60
w/o ATP
15 30 60
min
Mw (kDa)
170
100
55
myosin -Ubn
*E1
myosin-Ub1
myosin
c
myosin + UNC-45
myosin
Mw (kDa)
Inp Elu Inp Elu
120
100
85
70
UNC-45
myosin
d
myosin + UNC-45 4°C
myosin + UNC-45 27°C
w/o ATP
15 30 60
w/o ATP
15 30 60
min
Mw (kDa)
170
100
55
myosin -Ubn
*E1
myosin-Ub1
myosin
e
myosin
myosin + UNC-45ΔUCS
myosin + UNC-45
myosin + UNC45 + UFD-2 (60 min)
w/o ATP 15 30 60
w/o ATP 15 30 60
w/o ATP 15 30 60
min
Mw (kDa)
180
100
70
myosin -Ubn
*E1
-myosin-Ub1
-myosin
CHN-1
UFD-2 + UNC-45
CHN-1 + UNC-45
CHN-1 + UNC-45ΔUCS
CHN-1, w/o UNC-45
myosin-Ubn signal (arbitrary units)
1×10⁷
5×10⁶
0
0
20
40
60
time (min)
f
μl standard
Mw (kDa)
12 8 6 4 2 1
wild-type
E781K
ΔUCS
ΔTPR
w/o ATP
180
100
70
myosin -Ubn
*E1
-myosin-Ub1
-myosin
HRP-signal intensity (arbitrary units)
8×10⁰⁶
6×10⁶
4×10⁶
2×10⁶
0
E781K
ΔUCS
ΔTPR
w/o ATP
wild-type
R² = 0.99
0
5
10
μl of standard

Supplementary Figure 6. Poly-ubiquitination of *C. elegans* myosin by UFD-2

(**a**) SEC profile and SDS-PAGE gel analysis of myosin preincubated for 60 min at 4°C or 27°C are shown. (**b**) Ubiquitination of the purified UNC-45:myosin complex by UFD-2 using native or methylated ubiquitin (MeUb). Western blot using antibodies against the His-tag demonstrates poly-ubiquitination of myosin by UFD-2. (**c**) Control for PD experiments shown in **Fig. 7c**, demonstrating that UNC-45 and myosin do not interact with the resin. (**d**) Ubiquitination of mixed UNC-45 and myosin samples preincubated at 4°C or 27°C, demonstrating that the UNC-45/UFD-2 (chaperone/E3 ligase) pair targets destabilized myosin. (**e**) Left panel: Reactions containing CHN-1, myosin and the indicated UNC-45 protein were incubated for 15, 30 and 60 min with ATP and a control reaction for 60 min without ATP, and analyzed by anti-His Western blot. A 60 min reaction time point of a ubiquitination assay containing UFD-2 is shown for comparison. Right panel: Quantification of ubiquitinated myosin. Ubiquitination signal observed without ATP was used for background subtraction and is displayed as time point 0 for every reaction mix. For comparison, the myosin poly-ubiquitination signal upon UFD-2 addition (orange) is shown. (**f**) Left panel: anti-His Western blot showing a dilution series (µl of standard reaction) of a ubiquitination reaction containing wild-type UNC-45, myosin and UFD-2, and reactions containing the indicated version of UNC-45 protein. Right panel: Quantification of ubiquitinated myosin. Known amounts of the ubiquitination reaction containing wild-type UNC-45, myosin and UFD-2 (µl of standard) were plotted against the quantified ubiquitination signal to generate a standard curve. All reactions are in the linear range of detection.

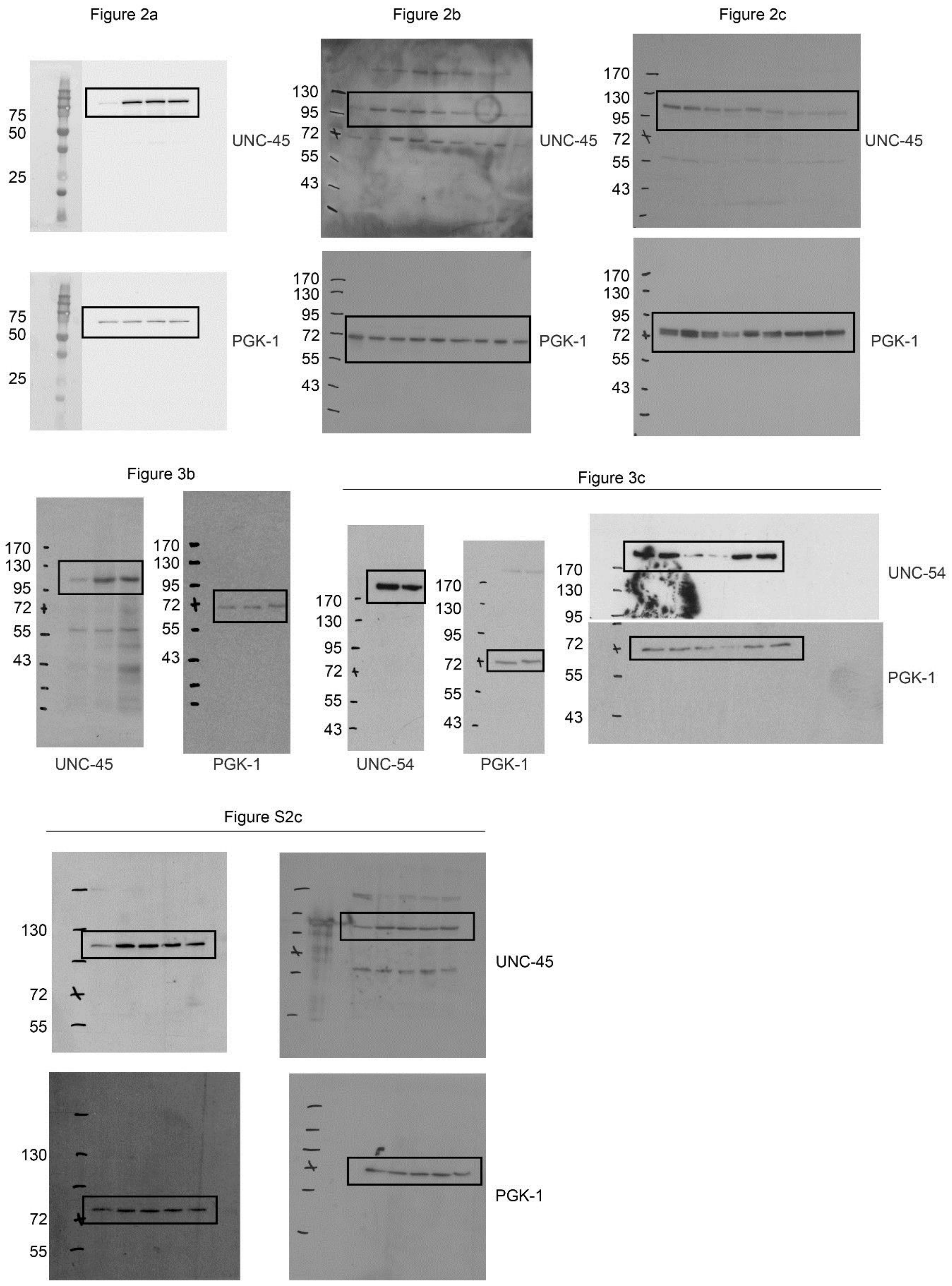

Supplementary Figure 7. Uncropped Western blots for the indicated Figures

SUPPLEMENTARY TABLES

Supplementary Table 1. Interaction partners of UFD-2 and UNC-45 identified by MS analysis

The most abundant proteins based on peak area in each immunoprecipitate are listed in order of abundance in the UFD-2 IP sample. ([a] number of identified peptides; [b] peptide spectra matches; ACT – actin; NMY – non-muscle myosin; TNT – troponin; MLC – myosin light chain). One replicate is shown for each IP (anti-control, anti-UFD-2 and anti-UNC-45).

Accession	Description	MW kDa	control IP				anti-UFD-2 IP				anti-UNC-45 IP			
			Cover-age	Pep.[a)]	PSMs[b)]	Area	Cover-age	Pep.	PSMs	Area	Cover-age	Pep.	PSMs	Area
Q6BEV4	**UFD-2**	**113,1**		**0**	**0**	**0,00E+00**	**56,08%**	**59**	421	**1,23E+08**	**17,26%**	**12**	14	**3,99E+06**
P90879	F49C12.9	34,7		0	0	0,00E+00	46,23%	16	48	4,04E+07	14,10%	4	4	1,63E+06
G5ECU5	F44E5.4, Hsp70 family heat shock protein	70,6	38,91%	25	50	6,66E+06	42,33%	28	84	2,32E+07	55,04%	34	95	4,01E+08
O45246	HSP-70	70,4	34,21%	19	40	6,66E+06	36,24%	22	42	2,32E+07	55,37%	28	66	4,01E+08
Q95ZL1	ACT-4	37,3	33,73%	9	34	2,52E+06	33,73%	9	26	2,74E+06	94,28%	30	348	7,04E+09
G5EF87	SWSN-1, putative component of the SWI/SNF complex	85,4		0	0	0,00E+00	31,69%	24	36	2,62E+06	2,66%	2	2	1,43E+06
O45815	ACT-5	41,8	25,07%	9	16	2,57E+06	25,07%	9	13	1,79E+06	72,00%	26	223	4,94E+09
G5EG62	**UNC-45**	**107,4**	**2,50%**	**1**	1	6,19E+04	**25,70%**	**22**	25	**1,56E+06**	**57,23%**	**60**	218	**7,93E+08**
E2JL06	OLA-1, ortholog of human Obg-like ATPase 1	13,9		0	0	0,00E+00	50,40%	6	8	1,26E+06	57,60%	6	8	7,65E+06
K8FDX2	LEV-11, tropomyosin	33,0	36,97%	11	14	3,85E+05	38,38%	11	15	1,15E+06	72,18%	37	287	3,20E+09
Q21000	MYO-5	226,9	6,03%	12	17	4,61E+05	1,22%	5	9	9,23E+05	62,16%	141	275	6,53E+08
B6EU49	Alkali myosin light chain long isoform	17,1	50,33%	7	8	1,97E+05	58,17%	8	10	5,49E+05	96,73%	21	166	1,69E+09
Q965K5	DNC-3	19,4		0	0	0,00E+00	38,01%	5	6	3,36E+05		0	0	0,00E+00
G5EF37	PAT-10, body wall muscle troponin C	18,5	4,35%	1	1	8,74E+04	4,35%	1	1	1,85E+05	49,69%	8	21	1,77E+08
Q20641	NMY-1	229,2	6,62%	11	12	9,15E+04	5,91%	10	10	1,41E+05	63,88%	155	427	2,91E+08
Q6LD30	Unc-87	39,7	4,20%	1	1	8,09E+04	4,20%	1	1	1,05E+05	74,79%	27	92	5,10E+08
B3WFV3	UNC-15, paramyosin	63,7	18,44%	9	10	9,57E+04	6,87%	3	3	4,21E+04	69,98%	54	143	4,29E+08
Q9XVI9	MLC-5	16,0		0	0	0,00E+00		0	0	0,00E+00	84,51%	11	24	1,89E+08
G5EBY3	NMY-2	231,1	6,24%	12	13	6,18E+04	0,50%	1	1	0,00E+00	61,56%	158	279	9,86E+07
O44556	TNT-4	40,7	2,31%	1	1	0,00E+00		0	0	0,00E+00	40,35%	14	20	2,19E+07
Q69Z12	MLC-7	17,3		0	0	0,00E+00		0	0	0,00E+00	76,47%	9	18	2,00E+07
H2KYL7	TNT-3	41,4	2,29%	1	1	0,00E+00		0	0	0,00E+00	28,57%	10	10	1,96E+07
Q21201	MLC-6	16,3		0	0	0,00E+00		0	0	0,00E+00	68,53%	11	12	9,34E+06

Supplementary Table 2. Ubiquitination sites within UNC-45 targeted by UFD-2 (identified by MS)

replicate / Ub sites[a)]	(1)	(2)	(3)	(4)
**TPR domain**				
K141	x			
**Central domain**				
K155	x			x
**UCS domain**				
K620		x	x	x
K629				x
K637	x	x	x	
K706		x	x	
K713	x	x	x	
K717	x			
K914	x			
K938	x	x	x	
K943	x			

a) Residues ubiquitinated by CHN-1: K20, K82, K115, K141, K147, K155, K250, K305, K385, K479, K497, K517, K614, K620, K629, K637, K706, K713, K717, K861, K914, K938, K943

Supplementary Table 3. Data collection and refinement statistics for the UNC-45_{717} structure

Space group	$P6_122$
Cell dimensions<br>*a*, *b*, *c* (Å)	 86.2, 86.2, 716.5
**Data collection**	
Resolution (Å)[a]	50-3.8 (3.87-3.80)
R_{sym}(%)	8.5 (36.7)
I /sigma(I)	15.9 (3.8)
Completeness (%)	93.5 (97.9)
Redundancy	4.4 (4.7)
**Refinement**	
Resolution (Å)	29.4-3.8
No. of reflections	15213
R_{work}/R_{free}(%)	29.8/31.9
No. atoms (protein)	5994
B-factor (protein)	184
rms deviations	
Bond lengths (Å)	0.006
Bond angles (º)	0.8
Ramachandran statistics	
favored	89.9%
outliers	2.2%

[a](highest resolution shell is shown in parentheses)

Supplementary Table 4. UFD-2 and UNC-45 peptides used for parallel reaction monitoring (PRM)

	Peptide sequence	Mass [m/z]	CS [z]	Start [min]	End [min]	(N)CE	(N)CE type
**UFD-2**	FVLLSQDGSR	561.30111	2	63.84	78.84	27	NCE
	LLEDTVSNVFLR	703.38792	2	95.34	110.34	27	NCE
	EDFLPTPSEK	581.78496	2	62.05	77.05	27	NCE
	LNTVSGFER	511.76691	2	45.49	60.49	27	NCE
	SPFLVSK	389.2289	2	45.85	60.85	27	NCE
	TPVLGER	386.2216	2	29.09	44.09	27	NCE
**UNC-45**	ALEFDGADVK	532.76657	2	59.45	74.45	27	NCE
	GIVEVLQR	457.27691	2	61.68	76.68	30	NCE
	ALYDSEDPTVK	619.30098	2	47.98	62.98	27	NCE
	FLLETEK	440.24474	2	55.25	70.25	27	NCE
	NALELIAR	450.26908	2	66.02	81.02	27	NCE

Supplementary Table 5. Sequences of codon-optimized protein expression constructs

C. elegans UNC-45
ATGGTTGCACGTGTTCAGACAGCCGAAGAAATTCGTGATGAAGGTAATGCAGCAGTGAAAGATCAGGATTATATCAAAGCCGATGA<br>ACTGTATACCGAAGCACTGCAGCTGACCACCGATGAAGATAAAGCACTGCGTCCGGTTCTGTATCGTAATCGTGCAATGGCACGTC<br>TGAAACGTGATGATTTTGAAGGTGCACAGAGCGATTGTACCAAAGCACTGGAATTTGATGGTGCAGATGTTAAAGCCCTGTTTCGT<br>CGTAGCCTGGCACGTGAACAGCTGGGTAATGTTGGTCCGGCATTTCAGGATGCAAAAGAAGCCCTGCGTCTGAGCCCGAATGATAA<br>AGGTATTGTTGAAGTTCTGCAGCGTCTGGTGAAAGCCAATAACGATAAAATCAAACAGACCACCAGTCTGGCCAATAAAGTTACCG<br>ATATGGAAAAACTGGCATTTCGTGGTGAAGCAAAAGATACCGAACAGAAAATGACCGCACTGAATAATCTGCTGGTTCTGTGTCGT<br>GAAAGCGAAAGCGGTGCAACCGGTGTTTGGAATCAGGGTGCACTGGTTCCGTTTGTTCTGAATCTGATTAATGATGCCAGCGAAAA<br>TGAAGAAGTTACCGTTACCGCAATTCGCATTCTGGATGAAACCATTAAAAACAGCGTTCGCTGCATGAAATTTCTGGCAATGCATG<br>ATCCGGATGGTCCGAAAAGCGTTCGTTTTGTTTGTCGTCTGATGTGCAAAAAAAGCACCAAAGATTTTGTTGATGCCACCGGTATT<br>CTGGTTCAGCGTGTGTTTAATGCAATGGCCAAAATGGATCGTCAGAAAGAAATGAAACCGGATCCTGAAGTTGCAGAAGCAAACAA<br>AATTTGGATTATTCGTGTTCTGCTGGAACTGCAAGAAATGCTGCAGGATCCGAAAGTTGGTGCAGTGCAGCGTGAAACCTGTATTG<br>ACCTGTTTCTGAAAAATCTGATGCACATGGATGGTGGTATTCCGCGTGGTTGGAGCTGGAAATTTGTTGAAGAACGTGGTCTGCTG<br>GCCCTGCTGGATGTTGCAAGCCAGATTCCGGAACTGTGTGAATATCCGGTTAGCGCAGAAACCCGTCAGCATGTTGCAATTTGTCT<br>GCAACGCCTGGAAGAAGATATGGTCTTTGATACCAAACGCACCATCTTCAAAGAAAAAGTGGATATGTTTTTTAACGCCCTGATTA<br>GCCGTTGCACCAATGATGATGAAGGCCATAAATATCGCATTAAACTGAGCTGCTTTCTGATTACCATGCTGCAAGGTCCGGTTGAT<br>ATTGGCATTAATCTGATCACAAATGATCAGCTGACGCCGATTATGCTGGAAATGGCAGCAAGCCAGGATCACCTGATGCAGGGTAT<br>TGCAGCAGAACTGATTGTTGCAACCGTTAGCAAACATGAACGTGCGATTAATATGCTGAAAGTGGGTATTCCGGTGCTGCGTGCAC<br>TGTATGATAGCGAAGATCCGACCGTTAAAGTTCGTGCCCTGGTTGGTCTGTGTAAAATTGGTGCAGCCGGTGGTGATGATATTAGC<br>AAAGCAACCATGAAAGAAGAAGCCGTTATTAGCCTGGCCAAAACCTGCAAAAAATTCCTGCTGGAAACCGAGAAATACAGCGTTGA<br>TATTCGTCGTTATGCATGTGAAGGTCTGAGTTATCTGAGCCTGGATGCCGATGTGAAAGAATGGATTGTTGATGATAGCCTGCTGC<br>TGAAAGCCCTGGTGCTGCTGGCGAAAAAAGCCGGTGCACTGTGTGTTTATACCCTGGCAACCATTTATGCAAATCTGAGCAATGCC<br>TTTGAGAAACCGAAAGTGGATGAAGAAATGGTTAAACTGGCACAGTTTGCCAAACATCATGTTCCGGAAACCCATCCGAAAGATAC<br>GGAAGAATATGTGGAAAAACGTGTTCGCGCACTGGTTGAAGAGGGTGCAGTTCCGGCATGTGTTGCAGTTAGCAAAACCGAATCAA<br>AAAATGCCCTGGAACTGATCGCACGCAGCCTGCTGGCATTTGCAGAATATGAAGATCTGCGTGGTCGTATTATTGCCGAAGGTGGC<br>ACCGTTCTGTGCCTGCGCCTGACCAAAGAAGCATCAGGCGAAGGCAAAATCAAAGCAGGTCATGCAATTGCGAAACTGGGTGCAAA<br>AGCAGATCCGATGATTAGCTTTCCGGGTCAGCGTGCCTATGAAGTTGTTAAACCGCTGTGTGATCTGCTGCATCCAGATGTTGAAG<br>GTAAAGCAAATTATGACTCACTGCTGACCCTGACCAATCTGGCAAGCGTTAGCGATAGCATTCGCGGTCGTATTCTGAAAGAGAAA<br>GCAATTCCGAAAATCGAAGAGTTTTGGTTCATGACCGATCACGAACATCTGCGTGCAGCCGCAGCCGAACTGCTGCTGAATCTGCT<br>GTTTTTTGAAAAATTCTACGAGGAAACCGTGGCACCGGGTACAGATCGCCTGAAACTGTGGGTGCTGTATAGTGCAGAAGTGGAAG<br>AGGAACGTCTGAGTCGTGCAAGCGCAGCCGGTTTTGCAATTCTGACCGAAGATGAAAATGCATGTGCACGTATTATGGACGAAATC<br>AAAAGCTGGCCTGAAGTGTTTAAAGATATCGCCATGCATGAAGATGCCGAAACCCAGCGTCGTGGTCTGATGGGTATTGCCAATAT<br>TATGCATAGCAGCAATAAACTGTGCAGCGAAATTGTTAGCAGCGAAGTTTTTCGTGTGCTGGTTGCCGTTACCAAACTGGGTACAA<br>TTAATCAAGAACGTGCAGGTAGCACCGAACAGGCAAAACGCGGTCTGGAAGCCGCAGAAAAATTTGGTCTGATTAAAGCAACCGAT<br>CGCGAAATTTATGAACGCGAAAATCAGATGAGCACCATTCAAGAA

HsUNC-45b

```
ATGGCAGAAGTTGAAGCAGTACAGCTGAAAGAAGAAGGTAATCGTCATTTTCAGCTGCAGGATTATAAAGCAGCAACCAATAGCTA
TAGCCAGGCACTGAAACTGACCAAAGATAAAGCACTGCTGGCAACCCTGTATCGTAATCGTGCAGCATGTGGTCTGAAAACCGAAA
GCTATGTTCAGGCAGCAAGTGATGCAAGCCGTGCAATTGATATTAACAGCAGCGATATCAAAGCACTGTATCGTCGTTGTCAGGCA
CTGGAACATCTGGGTAAACTGGATCAGGCATTTAAAGATGTTCAGCGTTGTGCAACCCTGGAACCGCGTAATCAGAATTTTCAAGA
AATGCTGCGTCGTCTGAATACCAGCATTCAAGAAAAACTGCGTGTTCAGTTTAGCACCGATAGCCGTGTTCAGAAAATGTTTGAAA
TTCTGCTGGATGAAAACAGCGAAGCCGATAAACGTGAAAAAGCAGCCAATAATCTGATTGTTCTGGGTCGTGAAGAAGCAGGCGCA
GAAAAAATCTTTCAGAATAATGGTGTTGCACTGCTGCTGCAGCTGCTGGATACCAAAAAACCGGAACTGGTTCTGGCAGCAGTTCG
TACCCTGAGCGGTATGTGTAGCGGTCATCAGGCACGTGCAACCGTTATTCTGCATGCCGTTCGTATTGATCGTATTTGTAGCCTGA
TGGCCGTTGAAAATGAAGAAATGAGCCTGGCAGTTTGTAATCTGCTGCAGGCAATTATTGATAGCCTGAGTGGTGAAGATAAACGC
GAACATCGTGGTAAAGAAGAGGCACTGGTACTGGACACAAAAAAAGATCTGAAACAAATCACCAGCCATCTGCTGGACATGCTGGT
TAGCAAAAAAGTTAGCGGTCAGGGTCGTGATCAGGCCCTGAATCTGCTGAATAAAAACGTTCCGCGTAAAGATCTGGCCATTCATG
ATAATAGCCGTACCATTTATGTGGTGGATAATGGTCTGCGTAAAATCCTGAAAGTTGTTGGTCAGGTTCCGGATCTGCCGAGCTGT
CTGCCGCTGACCGATAATACCCGTATGCTGGCAAGCATTCTGATCAACAAACTGTATGATGATCTGCGTTGTGATCCTGAACGTGA
TCATTTTCGTAAAATCTGCGAAGAATACATCACCGGCAAATTTGATCCGCAGGATATGGATAAAAATCTGAACGCAATTCAGACCG
TTAGCGGTATTCTGCAGGGTCCGTTTGATCTGGGCAATCAACTGCTGGGTCTGAAAGGTGTTATGGAAATGATGGTTGCACTGTGT
GGTAGCGAACGTGAAACCGATCAGCTGGTTGCAGTTGAAGCCCTGATTCATGCAAGCACCAAACTGAGCCGTGCCACCTTTATTAT
CACCAATGGTGTGAGCCTGCTGAAACAAATTTACAAAACCACCAAAAACGAGAAAATCAAAATTCGCACCCTGGTTGGTCTGTGCA
AACTGGGTAGTGCCGGTGGCACCGATTATGGCCTGCGTCAGTTTGCAGAAGGTAGCACCGAAAAACTGGCAAAACAGTGTCGTAAA
TGGCTGTGCAATATGAGCATTGATACCCGTACCCGTCGTTGGGCAGTGGAAGGTCTGGCATATCTGACCCTGGATGCAGATGTTAA
AGATGATTTTGTTCAGGATGTTCCGGCACTGCAGGCCATGTTTGAACTGGCCAAAGCAGGCACCAGCGATAAAACCATTCTGTATA
GCGTTGCAACCACCCTGGTGAATTGTACCAATAGTTATGATGTGAAAGAGGTGATTCCTGAACTGGTGCAGCTGGCAAAATTTAGC
AAACAGCATGTTCCGGAAGAACACCCGAAAGATAAAAAAGATTTTATCGATATGCGCGTGAAACGTCTGCTGAAAGCCGGTGTTAT
TAGCGCACTGGCATGTATGGTTAAAGCAGATAGCGCCATTCTGACCGATCAGACCAAAGAGCTGCTGGCTCGCGTTTTTCTGGCCC
TGTGTGATAATCCGAAAGATCGTGGCACCATTGTTGCACAGGGTGGTGGTAAAGCGCTGATTCCGCTGGCCCTGGAAGGCACCGAT
GTTGGTAAAGTTAAAGCCGCACATGCCCTGGCAAAAATTGCAGCAGTTAGCAATCCGGATATTGCATTTCCGGGTGAACGTGTTTA
TGAAGTTGTTCGTCCGCTGGTTCGCCTGCTGGACACCCAGCGTGATGGTCTGCAGAATTATGAAGCGCTGCTGGGCCTGACCAATC
TGAGCGGTCGTAGCGATAAACTGCGTCAGAAAATCTTCAAAGAACGTGCACTGCCGGATATCGAAAACTATATGTTTGAGAATCAT
GATCAGCTGCGTCAGGCAGCCACCGAATGTATGTGTAATATGGTTCTGCATAAAGAAGTGCAAGAACGTTTTCTGGCAGATGGTAA
TGATCGTCTGAAACTGGTTGTTCTGCTGTGCGGTGAAGATGATGATAAAGTTCAGAATGCAGCAGCCGGTGCACTGGCCATGCTGA
CCGCAGCACATAAAAAACTGTGTCTGAAAATGACCCAGGTTACCACCCAGTGGCTGGAAATCCTGCAACGTCTGTGTCTGCATGAT
CAACTGAGCGTTCAGCATCGTGGCCTGGTTATTGCCTATAACCTGCTGGCAGCCGATGCAGAACTGGCGAAAAAACTGGTGGAAAG
TGAACTGCTGGAAATTCTGACAGTTGTGGGTAAACAAGAACCGGATGAAAAAAAAGCAGAAGTGGTTCAGACCGCACGTGAATGTC
TGATTAAATGTATGGATTATGGCTTTATCAAACCGGTGAGC
```

Supplementary Table 6. Primers used in this study

Construct	Forward primer	Reverse primer
UCS-His6 in pGEX-6P	CCGGAATTCGCCGTTATTAGCCTGGCCAAA	GATCCTCGAGTTAATGGTGGTGGTGATGATGTCCTCCTTCTTGAATGGTGCTCAT
DeltaUCS-Strep in pET21a	GGAATTCCATATGGTTGCTCGAGTACAGACTGCG	ATAAGAATGCGGCCGCTTATTTTTCGAACTGCGGGTGGCTCCAAGCGCTTTTCATCGTTGCTTTCGAAAT
DeltaUCS His	GTTTAACTTTAAGAAGGAGATATACCATGGTTGCTCGAGTACAGACTGC	TCAGTGGTGGTGGTGGTGGTGTTTCATCGTTGCTTTCGAAATGTCGTC
DeltaTPR-His in pET21a	GGAATTCCATATGACCACTTCACTGGCTAATAAG	ATAAGAATGCGGCCGCTTCCTGAATGGTGCTCATTTG
luciferase-Strep in pET21a	GGAATTCCATATGGAAGACGCCAAAAACATAAAG	GATCCTCGAGTTATTTTTCGAACTGCGGGTGGCTCCACAATTTGGACTTTCCGCCCTTCTT
CHN-1 in pET-SUMO	CTAGCTAGCATGTCAAGCGGCGCCGAACAACATAAT	GATCTCGAGTTAAACCATGCCTCCGGGTTCATA
UFD2 in pET-SUMO and pET28a	GCAGCCATGGCAATGATTGAAGACGAGAAAGCA	GATCCTCGAGTTATTTCTTTGAATTTCTTTTCTGGCA
UFD-2-Strep in pCoofy	GTTTAACTTTAAGAAGGAGATATACCATGATTGAAGACGAGAAAGCAGGCTTG	CTGCGGGTGGCTCCAAGCGCTTTTCTTTGAATTTCTTTTCTGGCAAATCCATTC
UNC-45 E781K-Strep in pCoofy	GTTTAACTTTAAGAAGGAGATATACCATGGTTGCTCGAGTACAGACTGC	CTGCGGGTGGCTCCAAGCGCTTTCCTGAATGGTGCTCATTTGATTTTCG
DeltaTPR-Strep in pCoofy	GTTTAACTTTAAGAAGGAGATATACCATGACCACTTCACTGGCTAATAAGGTAACTG	CTGCGGGTGGCTCCAAGCGCTTTCCTGAATGGTGCTCATTTGATTTTCG
myosin in pACEBac1	CGGATCCCGGTCCGAAACCATGGAGCACGAGAAGGACCCAG	CCCCAGAACATCAGGTTAATGGCGCTAATGATGGTGGTGATGGTGGAGC
UNC-45 in pIDC derivative	CGGATCCCGGTCCGAAACCATGGTTGCTCGAGTACAGACTGC	CTGCGGGTGGCTCCAAGCGCTTTCCTGAATGGTGCTCATTTGATTTTCG

Side directed mutagenesis - construct	Forward primer
UNC-45 E781K	ATTCCAAAGATTGAGAAATTCTGGTTTATGACG
UNC-45 K637R	GAATATGTTGAACGCCGAGTGAGAGCT
UNC-45 K914R	GTCGCCGTCACACGCCTCGGCACTATC
UNC-45 K704, K706R	GGAGAGGGACGCATCCGCGCGGGACAT
UNC-45 K713, K717R	ATTGCTCGCCTTGGGGCTCGCGCGGAT
UNC-45 K938, 943R	GCCGAGCGCTTTGGACTGATTCGCGCGACG
